# Transcriptome and Co-expression Network Analyses Reveal Differential Gene Expression and Pathways in Response to Severe Drought Stress in Peanut (*Arachis hypogaea* L.)

**DOI:** 10.3389/fgene.2021.672884

**Published:** 2021-04-30

**Authors:** Nannan Zhao, Shunli Cui, Xiukun Li, Bokuan Liu, Hongtao Deng, Yingru Liu, Mingyu Hou, Xinlei Yang, Guojun Mu, Lifeng Liu

**Affiliations:** State Key Laboratory for Crop Improvement and Regulation, Hebei Agricultural University, Baoding, China

**Keywords:** peanut (*Arachis hypogaea* L.), RNA-seq, WGCNA, drought stress, differentially expressed genes

## Abstract

Drought is one of the major abiotic stress factors limiting peanut production. It causes the loss of pod yield during the pod formation stage. Here, one previously identified drought-tolerant cultivar, “L422” of peanut, was stressed by drought (35 ± 5%) at pod formation stage for 5, 7, and 9 days. To analyze the drought effects on peanut, we conducted physiological and transcriptome analysis in leaves under well-watered (CK1, CK2, and CK3) and drought-stress conditions (T1, T2, and T3). By transcriptome analysis, 3,586, 6,730, and 8,054 differentially expressed genes (DEGs) were identified in “L422” at 5 days (CK1 vs T1), 7 days (CK2 vs T2), and 9 days (CK3 vs T3) of drought stress, respectively, and 2,846 genes were common DEGs among the three-time points. Furthermore, the result of weighted gene co-expression network analysis (WGCNA) revealed one significant module that was closely correlated between drought stress and physiological data. A total of 1,313 significantly up-/down-regulated genes, including 61 transcription factors, were identified in the module at three-time points throughout the drought stress stage. Additionally, six vital metabolic pathways, namely, “MAPK signaling pathway-plant,” “flavonoid biosynthesis,” “starch and sucrose metabolism,” “phenylpropanoid biosynthesis,” “glutathione metabolism,” and “plant hormone signal transduction” were enriched in “L422” under severe drought stress. Nine genes responding to drought tolerance were selected for quantitative real-time PCR (qRT-PCR) verification and the results agreed with transcriptional profile data, which reveals the reliability and accuracy of transcriptome data. Taken together, these findings could lead to a better understanding of drought tolerance and facilitate the breeding of drought-resistant peanut cultivars.

## Introduction

Peanut (*Arachis hypogaea* L.) is one of the most important oil crops and economic crops in the world. It is a vital vegetable oil and protein source and is widely distributed in the tropical and subtropical regions. Drought is one of the most severe abiotic stresses that affects plant growth and development and causes constraint to agricultural productivity (Shao et al., 2009; Osakabe et al., 2014). Drought not only severely limits the growth and production of peanuts, but also causes higher levels of aflatoxin infection (Girdthai et al., 2010; Jeyaramraja et al., 2018). It has become an important limiting factor to improve the yield and quality of peanuts. Therefore, improving the drought resistance of varieties has become an important goal of peanut breeding.

Timing, duration, and severity of drought are important factors affecting peanut yield and quality (Rao et al., 1989; Dang et al., 2013). In general, the form of peanut is slightly drought-resistant, but in some specific periods, water shortage seriously affects the yield of peanut. In the pod formation stage, drought can severely reduce yield of peanut because it can largely decrease the number and fullness of pods (Rao et al., 1985; Koolachart et al., 2013; Yang et al., 2019). Therefore, understanding the molecular basis of drought response at pod formation stage is essential in peanut breeding programs to improve pod yield.

Plants have evolved complex molecular, physiological, and biochemical processes to cope with the effects of drought (Ramachandra Reddy et al., 2004; Shinozaki and Yamaguchi-Shinozaki, 2007; Shao et al., 2009; Osakabe et al., 2014). For example, drought stress causes the production of reactive oxygen species (ROS), and excessive ROS would lead to oxidative stress, inhibit plant growth, and even cause cell death. The key enzymes in plants can change under stress conditions, including superoxide dismutase (SOD), catalase (CAT), peroxidase (POD), ascorbate peroxidase (APX), glutathione reductase (GR), and so on, to involve themselves in the detoxification of ROS (Reddy et al., 2004). Also, plant response to drought stress includes osmotic regulation and hormone regulation (Shao et al., 2009; Gong et al., 2020). For example, Malondialdehyde (MDA) is the product of lipid peroxidation, and its dynamic accumulation in plant cells indicates the degree of membrane damage (Deng et al., 2019). Soluble sugar and protein act as osmoregulatory substances to protect plants from stress (Shao et al., 2009; Ozturk et al., 2020). In the molecular process, numerous functional genes and regulatory genes have been discovered under drought stress (Harb, 2016). For example, late embryogenesis abundant (LEA) proteins play a crucial role in protecting cells during dehydration (Hanin et al., 2011). Overexpression of *TaSnRK2.9* enhanced tobacco tolerance to drought and salt stresses through improved ROS scavenging ability (Feng et al., 2019). Transcription factors (TFs) also play a vital role in the response to drought stress, such as heat shock factor (HSF), basic helix-loop-helix (bHLH), NAC, and WRKY transcription factor families (Castilhos et al., 2014; Joshi et al., 2016; Duan et al., 2017; Guo et al., 2019; Manna et al., 2020; Zeng et al., 2020). Although there are many studies on drought resistance in plants, drought resistance is a complex trait controlled by a large number of genes, which has still not been fully elucidated and needs more investigation (Budak et al., 2015; Kumar et al., 2017).

Transcriptomic analysis is a highly efficient way to investigate genome function and the related important pathways (Mia et al., 2020). Many studies have been carried out using transcriptome analysis for drought stress in numerous crops (Ma et al., 2017; Zhu et al., 2019; Hao et al., 2020; Tiwari et al., 2021). A few studies have revealed many genes involved in drought stress in peanuts using transcriptome analysis (Zhao et al., 2018; Bhogireddy et al., 2020; Huang et al., 2020; Jiang et al., 2021). Nevertheless, the drought-related networks need to be further explained using transcriptome analysis due to the complexity of the relevant genetic pathways. With the recent development of bioinformatics, weighted gene co-expression network analysis (WGCNA) can be used for identifying genes with similar expression patterns that may participate in specific biological functions (Langfelder and Horvath, 2008). Our previous study has shown that “L422” is a drought tolerant cultivar (Zhao N. et al., 2020). Plants can preserve water through various anatomical features when subjected to drought, such as reducing leaf surface area by leaf rolling, folding, or shedding (Goche et al., 2020). Here, the transcriptional response of the leaves of “L422” to severe drought was analyzed at the pod formation stage by using RNA sequencing (RNA-seq). Further, differential gene expression in multiple crucial signaling pathways involved in plant drought stress was analyzed from the module that was strongly correlated with drought stress and physiological data using WGCNA. These findings will provide a valuable resource for the study of drought resistance in peanut and lay a foundation for further targeted research on drought resistance genes.

## Materials and Methods

### Plant Materials and Drought Treatment

Peanut cultivar “L422” was a drought tolerant cultivar based on a previous study (Zhao N. et al., 2020). “L422” was planted in rainout shelters in Baoding, China (115°E, 38°N) in 2019, and confirmed again to be drought tolerant. In brief, “L422” (drought-resistant) (Zhao N. et al., 2020), “Huayu 23” (drought-sensitive) (Ding et al., 2017), “Huayu 25” (drought-resistant) (Zhang et al., 2011), and “L632” (drought-sensitive; data not shown) cultivars with different drought tolerances were planted in environmentally controlled rainout shelters (6 m × 8 m), with two water treatments (well-watered and drought) and three replicates. The relative soil water content (RSWC) was maintained at 70–75% until the plants reached the reproductive phase (pod formation stage). At the pod formation stage, the control group continued to be under well-watered conditions while the treatment group stopped irrigation until the RSWC of the soil decreased to 35%.

In the current study, we performed a transcriptomic analysis of “L422” at the pod formation stage under drought stress. Seeds were surface sterilized with 70% ethanol followed by thorough washing with sterile distilled water. After sterilizing, the seeds were soaked in deionized water at room temperature for 12 h. Subsequently, the seeds were placed in two layers of damp filter paper for 24 h in the dark to induce germination. Germinated seeds were planted in plastic pots (one seedling for each pot) in rainout shelters (Baoding, China) under well-watered conditions at 70–75% RSWC. The pots were 29.5 cm in diameter, 26.0 cm in ground diameter, and 23.5 cm in height. Then peanut seedlings under the same cultivation conditions were divided into control group and treatment group. The control group was well-watered continually, and irrigation was interrupted for the treatment group when peanuts entered the pod formation stage, which was 75 days after planting (DAP). Based on the RSWC (35 ± 5%) and phenotypic changes of the treatment group, fully expanded leaves from the main stem (Third nodal) of control (CK) and treatment (T) plants were sampled after 5 (80 DAP), 7 (82 DAP), and 9 (84 DAP) days of drought treatment, and then were immediately frozen in liquid nitrogen and stored at −80°C for subsequent analyses. Each treatment was replicated three times.

### Physiological Index Measurements

Phenotypic and physiological characterizations were determined for “L422” under well-watered and drought-stress conditions. The relative water content (RWC) was determined based on the method described by Galmes et al. (2007). Similarly, the relative electric conductivity of the peanut leaves was measured according to the method of Zhang et al. (2018). The MDA content, POD activity, soluble sugar, and soluble protein content of samples were measured using physiological assay kits (Suzhou Grace Biotechnolgy Co., Ltd, Jiangsu, China) referring to the manufacturers’ recommendations based on the methods of thiobarbituric acid-reactive-substances (TBARs), guaiacol colorimetric, anthrone colorimetric, and bicinchoninic acid (BCA), respectively. All processes were biologically and temporally repeated in three independent and parallel experiments. Student’s *t*-test was performed to calculate the *p*-values using GraphPad Prism software, version 8.01 (GraphPad Software, Inc., San Diego, CA, United States).

### Transcriptome Sequencing and *de novo* Assembly Analysis

The isolation of total RNA from non-stressed and stressed leaves of “L422” was optimized according to the instruction manual of the Trizol Reagent (Invitrogen, Carlsbad, CA, United States). RNA degradation and contamination were monitored on 1% agarose gels. The quality of the RNA was evaluated using Agilent 2100 Bioanalyzer (Agilent Technologies, Palo Alto, CA, United States), and 18 qualified RNA samples were used for RNA-seq analysis. A library was constructed using the NEBNext Ultra RNA Library Prep Kit for Illumina (NEB, Ipswich, MA, United States). The cDNA library construction and sequencing were carried out on the Illumina HiseqTM 2500 platform by Gene Denovo Biotechnology Co. (Guangzhou, China).

### Sequencing Reads Processing and Mapping

The quality of raw data (raw reads) was firstly processed by fastp (version 0.18.0) (Chen et al., 2018). In this step, clean data (clean reads) was obtained by removing reads containing adapters, more than 10% of unknown nucleotides (N), and more than 50% of low quality (*Q*-value ≤ 20) bases. Meanwhile, Q20 (99% base call accuracy), Q30 (99.9% base call accuracy), GC-content, and sequence duplication levels of the clean data were calculated. Qualified clean reads were then mapped to the peanut reference genome sequence (Tifrunner.gnm1.ann1.CCJH) using a spliced aligner HISAT2 software (version 2.2.4) (Kim et al., 2015). The mapped reads of each sample were assembled using StringTie (version 1.3.1) (Pertea et al., 2015). The gene expression level was normalized using the FPKM (Fragments per Kilobase of transcript per Million mapped reads) method. All the downstream analyses were based on high-quality clean data.

### Differential Expression Analysis

Differential gene expression analysis of the two groups was performed by DESeq2 (Love et al., 2014). The corrected *p*-values were used to control the false discovery rate (FDR). Genes were considered to be differentially expressed when the value of log2 Fold Change was >2 or <-2 with an FDR value below 0.01 between two groups. The Gene Ontology (GO) functions and Kyoto Encyclopedia of Genes and Genomes (KEGG) pathways enrichment analysis of differentially expressed genes (DEGs) were conducted using the hypergeometric test by comparing with the whole genome background. GO terms and KEGG pathways with FDR-corrected *p*-value ≤ 0.05 were regarded as significantly enriched in DEGs.

### Weighted Gene Co-expression Network Analysis

Weighted gene co-expression network analysis is a systems biology method for describing the correlation patterns among genes across multiple samples. This method aims to find clusters (modules) of highly correlated genes and relating modules to external sample traits (Zhang and Horvath, 2005). Co-expression networks were constructed using WGCNA (version 1.47) package in R (Langfelder and Horvath, 2008). After filtering non-varying or low-abundance (FPKM < 2) genes of samples (>70%), gene expression values were imported into WGCNA to construct co-expression modules using the automatic network construction function blockwise modules with default settings, except that the power is 10, TOMType is unsigned, mergeCutHeight is 0.75, and minModuleSize is 50. Genes were clustered into nine correlated modules.

### Gene Expression Validation

Nine genes with different expression profiles obtained by Illumina RNA-seq were randomly selected for validation by qPCR. Gene-specific primers were designed by Wcgene Biotech (Shanghai, China) (Supplementary Table 1). The Actin gene was used as housekeeping gene. Three biological and technical repetitions were used for each sample. The quantitative real-time PCR (qRT-PCR) was run on the ABI StepOnePlus instrument using Fast Super EvaGreen^®^ qPCR Master Mix (US Everbright^®^ Inc., China) according to the manufacturer’s instructions. The amplification program was set as follows: 95°C for 2 min followed by 45 cycles of 95°C for 5 s and 60°C for 1 min. All data from qRT-PCR amplification were calculated with 2^–△△CT^ method (Livak and Schmittgen, 2001).

## Results

### Physiological and Phenotypic Changes of Peanuts Under Drought Stress

To investigate the physiological responses of peanuts to water deficit, the physiological indexes were evaluated at the pod formation stage, including leaf RWC and relative electrical conductivity (Supplementary Figure 1). As shown in Supplementary Figure 1A, there were significant phenotypic changes in four peanut varieties. In terms of leaves, peanuts shriveled up under drought stress in “Huayu 23” and “Huayu 25,” but “Huayu 23” withered intensely. Although the leaves of “L632” did not wither like “Huayu 23” and “Huayu 25,” they turned yellow. However, the phenotypic change was not obvious in “L422.” The RWC of “L422,” “Huayu 23,” “Huayu 25,” and “L632” decreased to 32.3, 47.7, 34.6, and 44.0% under drought stress, respectively, as compared with the control (Supplementary Figure 1B). Relative electrical conductivity is widely used to measure the ability of plants to avoid or repair membrane damage. The relative electrical conductivity of “L422,” “Huayu 23,” “Huayu 25,” and “L632” increased by 107.6, 208.6, 108.8, and 167.8%, respectively (Supplementary Figure 1C).

**FIGURE 1:**
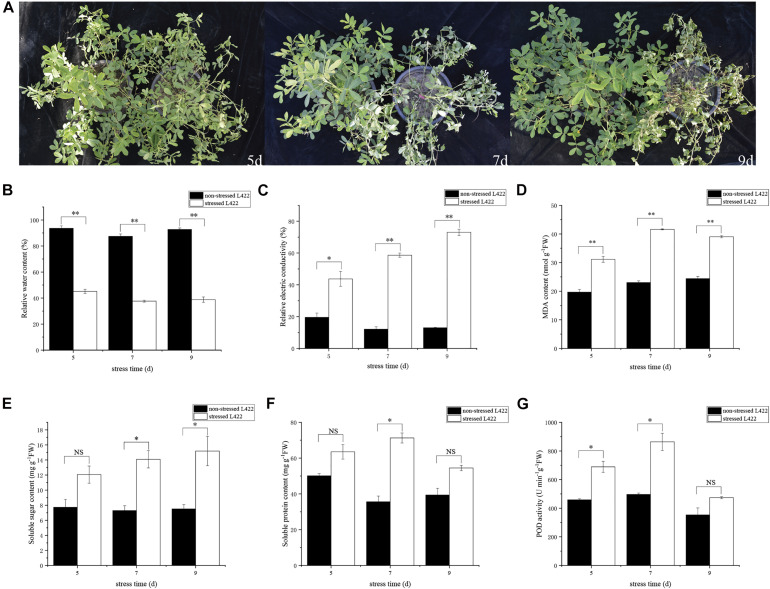
Phenotypic and physiological changes of “L422” under drought stress. **(A)** Phenotypic responses of “L422” to severe drought stress at three-time points (5, 7, and 9 days). The pots on the right and left correspond to the drought-treated and well-watered control peanut, respectively. The changes of the relative water content **(B)**, the relative electric conductivity **(C)**, MDA content **(D)**, soluble sugar content **(E)**, soluble protein content **(F)**, and POD activity **(G)** in leaves of “L422” under well-watered and drought conditions. Values are the mean ± standard deviation of three biological replicates. * and ** indicate the significant difference at 5% level and 1% level, respectively, “NS” indicates non-significant.

“L422” were planted in plastic pots and treated with drought at the pod formation stage. After 5 days of drought treatment, the leaves of “L422” began to shrivel up. After 7 and 9 days of drought treatment, “L422” showed distinct wilting (Figure 1A). Some physiological indicators’ response to drought stress were then measured. The leaf RWC decreased significantly (*p* < 0.01) with the increasing days of stress exposure (Figure 1B). Compared with the control, the RWCs of drought-treated leaves decreased to 51.8, 57.0, and 58.2% at 5, 7, and 9 days after drought treatment, respectively. The relative electrical conductivity of “L422” increased by 122.8, 383.6, and 460.9% in 5, 7, and 9 days, respectively (Figure 1C). Results of MDA content showed that the stressed group was significantly (*p* < 0.01) higher than the non-stressed group by 58.2, 80.8, and 59.9% under 5, 7, and 9 days with drought stress, respectively (Figure 1D). These data suggested that the leaves of “L422” were damaged under severe drought stress. As shown in Figure 1G, POD activity increased under severe drought stress but did not increase significantly at 9 days of drought stress. Compared with the control group, the soluble sugar content showed a trend of increase by 56.15, 93.5, and 102.3% at 5, 7, and 9 days with drought stress (Figure 1E). Additionally, the soluble protein content exhibited a greater increase at 7 days than 5 and 9 days of drought stress (Figure 1F).

### RNA Sequencing Analysis of “L422” Under Drought Stress

To investigate the key genes of peanuts in response to drought, the treated leaves of “L422” were sequenced. A total of approximately 94.04 million raw reads were generated from the 18 cDNA libraries (six samples × three replications) by RNA sequencing. The raw sequencing data had been deposited in NCBI under the accession number PRJNA706902. After deleting 0.60% of adapter sequences, and filtering 0.33% of low-quality reads and 0.00% of n-containing reads, 93.16 million high-quality clean reads were finally confirmed (Supplementary Table 2). The percentage of high-quality clean reads mapped to the peanut reference genome arahy.Tifrunner.gnm1.KYV3 ranged from 89.24 to 93.78% (Table 1). These results showed that the transcriptome sequencing quality was sufficient for further analyses.

**TABLE 1:** Summary of sequencing data for different samples.

Sample	RawData (bp)	CleanData (bp)	Q20 (%)	Q30 (%)	N (%)	GC (%)	Unmapped reads	Unique mapped reads	Total_Mapped (%)
T1-1	7443549900	7133966630	6948467942 (97.40%)	6642569327 (93.11%)	109097 (0.00%)	3178907415 (44.56%)	4133261 (8.54%)	33739797 (69.67%)	44291299 (91.46%)
T1-2	7478063700	7192087091	6990017761 (97.19%)	6680180461 (92.88%)	110950 (0.00%)	3213723611 (44.68%)	4183864 (8.62%)	33366382 (68.74%)	44353176 (91.38%)
T1-3	7402878300	7157768553	6963430531 (97.28%)	6662999707 (93.09%)	109804 (0.00%)	3202300203 (44.74%)	3607567 (7.50%)	33639294 (69.93%)	44493441 (92.50%)
T2-1	8447553300	8117926339	7905605355 (97.38%)	7574245766 (93.30%)	124397 (0.00%)	3653219812 (45.00%)	4953862 (9.04%)	37313003 (68.12%)	49820414 (90.96%)
T2-2	7438557600	7151027498	6957420272 (97.29%)	6655615104 (93.07%)	109852 (0.00%)	3205202420 (44.82%)	4642050 (9.62%)	32773043 (67.95%)	43588052 (90.38%)
T2-3	7893744900	7604749442	7407754289 (97.41%)	7095644448 (93.31%)	115653 (0.00%)	3397292779 (44.67%)	4724289 (9.20%)	35577300 (69.28%)	46630185 (90.80%)
T3-1	7752862500	7492206686	7286261792 (97.25%)	6960459720 (92.90%)	115313 (0.00%)	3344979970 (44.65%)	4628456 (9.20%)	34583523 (68.77%)	45661100 (90.80%)
T3-2	6797084400	6560746525	6379007254 (97.23%)	6102273974 (93.01%)	101097 (0.00%)	2903234585 (44.25%)	3928108 (8.95%)	30967665 (70.57%)	39952456 (91.05%)
T3-3	7047023400	6784517938	6600157401 (97.28%)	6311771245 (93.03%)	104954 (0.00%)	3050939041 (44.97%)	3824863 (8.37%)	31321183 (68.58%)	41849305 (91.63%)
CK1-1	8348989800	8055064749	7842051364 (97.36%)	7506055348 (93.18%)	123426 (0.00%)	3588914604 (44.55%)	4348583 (7.95%)	39021323 (71.38%)	50316235 (92.05%)
CK1-2	7285225200	7017227422	6813863910 (97.10%)	6494308099 (92.55%)	107792 (0.00%)	3143924964 (44.80%)	3916454 (8.25%)	33069245 (69.62%)	43581134 (91.75%)
CK1-3	7498422000	7201743917	7009836007 (97.34%)	6711677567 (93.20%)	111270 (0.00%)	3226974407 (44.81%)	3072703 (6.28%)	34751895 (71.03%)	45854949 (93.72%)
CK2-1	6220695600	6146941251	5605972285 (91.20%)	4903839074 (79.78%)	63350 (0.00%)	2753172797 (44.79%)	4392239 (10.76%)	30121830 (73.80%)	36424409 (89.24%)
CK2-2	7348115100	7030908860	6815994172 (96.94%)	6449075104 (91.72%)	12606 (0.00%)	3178348608 (45.21%)	3169138 (6.60%)	34231533 (71.28%)	44855020 (93.40%)
CK2-3	10131380700	9762527954	9504451596 (97.36%)	9096260361 (93.18%)	150432 (0.00%)	4368690192 (44.75%)	4127084 (6.22%)	47372258 (71.36%)	62261582 (93.78%)
CK3-1	8406291000	8118055249	7903244807 (97.35%)	7559991795 (93.13%)	125012 (0.00%)	3659974797 (45.08%)	3786157 (6.91%)	37898670 (69.16%)	51008683 (93.09%)
CK3-2	9171919200	8839433152	8615603298 (97.47%)	8257129252 (93.41%)	134137 (0.00%)	3972660094 (44.94%)	4132115 (6.91%)	41561446 (69.48%)	55688533 (93.09%)
CK3-3	8942134200	8616413360	8393324512 (97.41%)	8033373375 (93.23%)	132093 (0.00%)	3834640592 (44.50%)	3699547 (6.31%)	41809441 (71.36%)	54886911 (93.69%)

### Differentially Expressed Genes and qRT-PCR Validation

**FIGURE 2:**
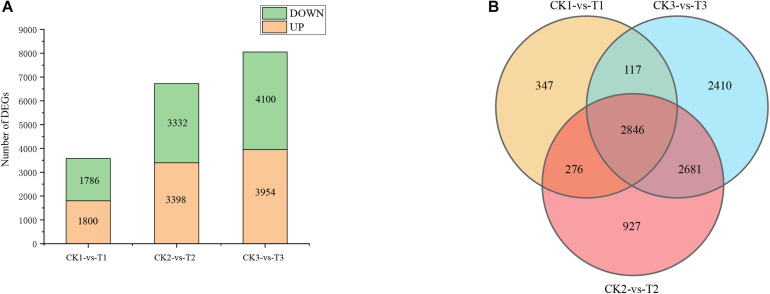
Difference analysis of gene expression by pairwise comparisons. **(A)** The number of DEGs induced by drought. **(B)** Venn diagram analysis of DEGs at the three-time points under severe drought stress. CK1-vs-T1: comparison between 5 days of drought and 5 days of well-watered condition; CK2-vs-T2: comparison between 7 days of drought and 7 days of well-watered condition; CK3-vs-T3: comparison between 9 days of drought and 9 days of well-watered condition.

Generally, a stringent threshold absolute log2 FC ≥ 2 and FDR < 0.01 was used to screen out DEGs. The number of DEGs after 5, 7, and 9 days of drought stress were 3,586, 6,730, and 8,054, respectively (CK1 vs T1, CK2 vs T2, and CK3 vs T3), and 2,846 genes were common DEGs among the three time points in “L422” (Figures 2A,B). After 5 days of drought treatment, 1,800 up-regulated DEGs and 1,786 down-regulated DEGs were identified. Of these DEGs after 7 days of drought treatment, 3,398 were up-regulated and 3,332 were down-regulated. After 9 days of continuous stress, the number of DEGs was the largest in the three treatment time points with 3,954 up-regulated genes and 4,100 down-regulated genes. On the whole, the number of up-regulated DEGs is higher than down-regulated DEGs, except for drought stress for 9 days. Together, the results revealed that the number of induced DEGs greatly increased with the continuation of drought stress time. All these DEGs were selected for further analysis.

**FIGURE 3:**
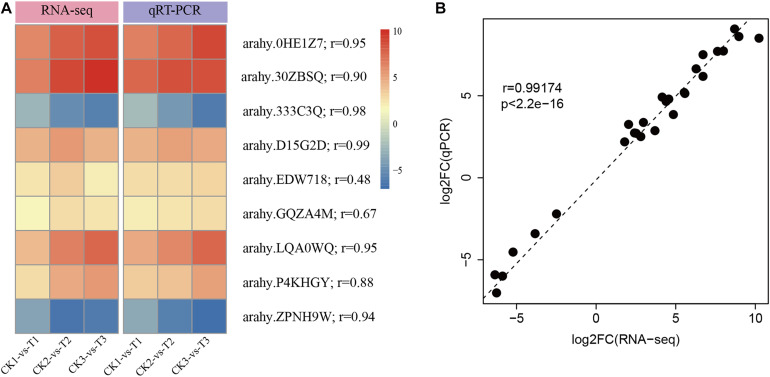
Confirmation of RNA-Seq results by quantitative real-time PCR (qRT-PCR). **(A)** The heatmap presentation of fold changes of nine DEGs obtained from RNA-seq analysis and qRT-PCR results. **(B)** Correlation between RNA-Seq expression profile and qRT-PCR results.

To experimentally confirm the results of RNA-Seq data, nine DEGs were randomly selected to perform qRT-PCR. As shown in Figure 3A, the selected DEGs had consistent expression patterns between RNA-Seq and qRT-PCR. The results showed a good correlation between the qRT-PCR results and the RNA-Seq results (*r* = 0.99, *p* < 2.2e-16, Figure 3B). This signifies that the RNA-seq data was of high-quality.

### Weighted Gene Co-expression Network Analysis Under Drought Stress

**FIGURE 4:**
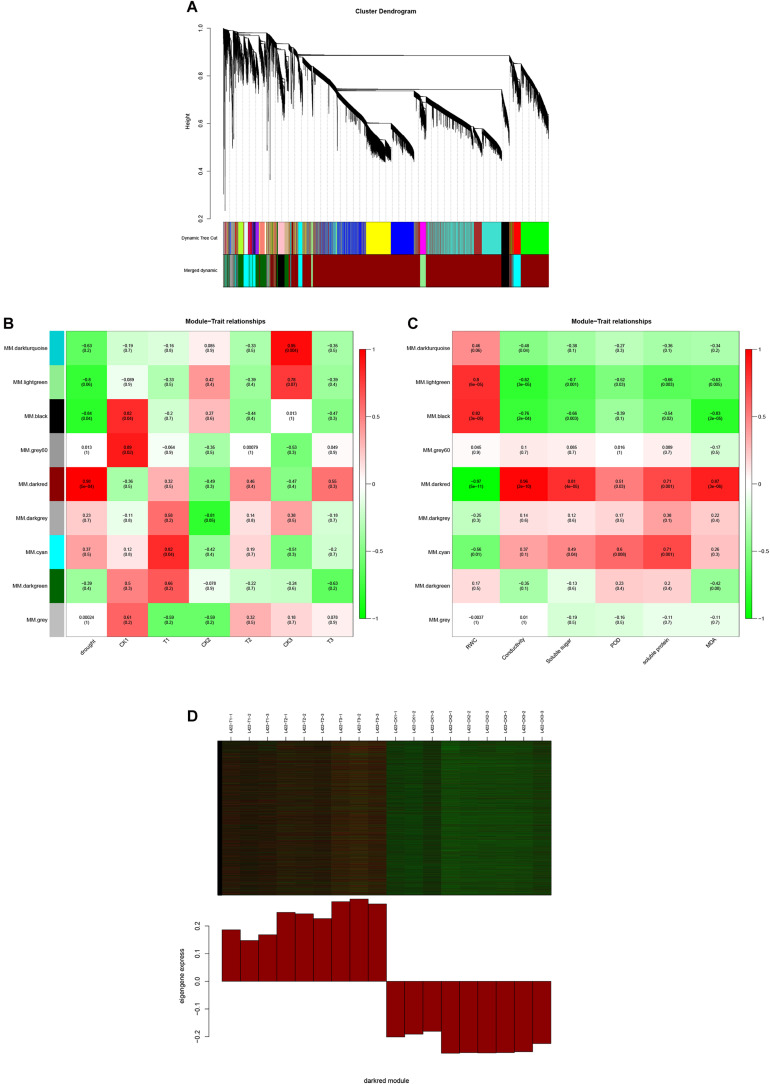
WGCNA of the transcripts changes in “L422.” **(A)** Hierarchical cluster tree shows nine modules of co-expressed genes in “L422.” Different modules are marked with different colors. Each leaf in the tree represents one gene. **(B)** Correlations of drought degree and samples with WGCNA modules. **(C)** Correlations of physiological indicators with WGCNA modules. The right color scale corresponds to module-trait correlation. Each row represents a specific module. The numbers in each cell represent the correlation coefficients and correlation significance levels (in parentheses). **(D)** Expression pattern of the genes and eigengenes of MM.darkred module. The heatmap was plotted using the log10 FPKM values.

To identify the expression of genes related to drought stress in peanut, a gene co-expression network was constructed using WGCNA. The 26,409 selected genes were assigned to nine merged co-expression modules (with various colors) (Figure 4A). As shown in Figure 4B, we successfully identified two modules significantly associated with drought stress for “L422” (*p* < 0.05). The MM.darkred module (*r* = 0.98, *p* = 5e-04) was positively correlated with resistance throughout the severe drought stress period, while the MM.black module (*r* = -0.84, *p* = 0.04) was negatively correlated with drought stress. Additionally, a module-trait relationships analysis was performed using module eigengenes and physiological data at each time point. As shown in Figure 4C, the MM.darkred module (*r* = -0.98, *p* = 2e-12) was negatively correlated with RWC under drought stress. In contrast, the MM.darkred module had a significant positive correlation with the RWC (*r* = 0.97, *p* = 5e-11), relative electrical conductivity(*r* = 0.96, *p* = 2e-10), soluble sugar (*r* = 0.81, *p* = 4e-05), POD (*r* = 0.51, *p* = 0.03), soluble protein(*r* = 0.71, *p* = 0.001), and MDA (*r* = 0.81, *p* = 3e-6). The identification of peanut genotype-specific modules in severe drought stress was particularly important. Based on the above results, the MM.darkred module was related to drought response, and was selected for further analysis.

### Enrichment Analysis of the Detected Co-expressed Modules

**FIGURE 5:**
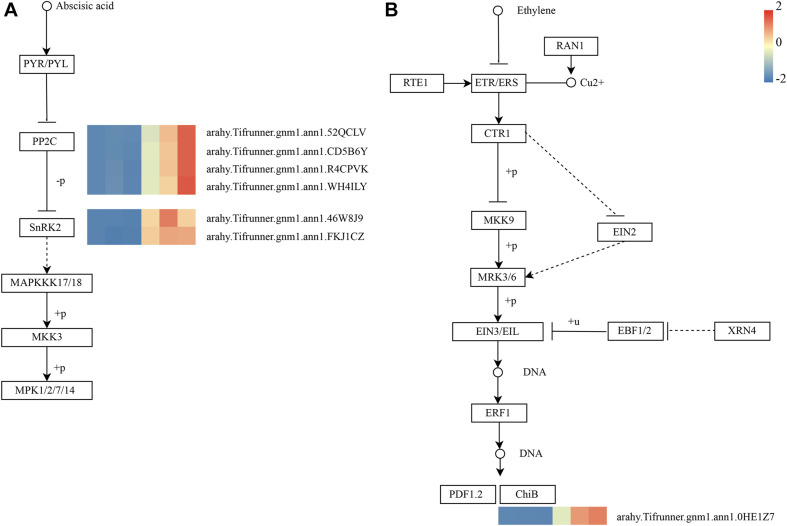
Drought-responsive genes of MM.darkred module in MAPK signaling pathway-plant. **(A)** ABA and **(B)** Ethylene signal transduction pathways. Relative expression levels are normalized based on the Z-score and shown as a color gradient from low (blue) to high (red). The columns in heat map are 5, 7, and 9 days of well-watered condition, and 5, 7, and 9 days of drought-treated condition under severe drought from left to right, respectively.

**FIGURE 6:**
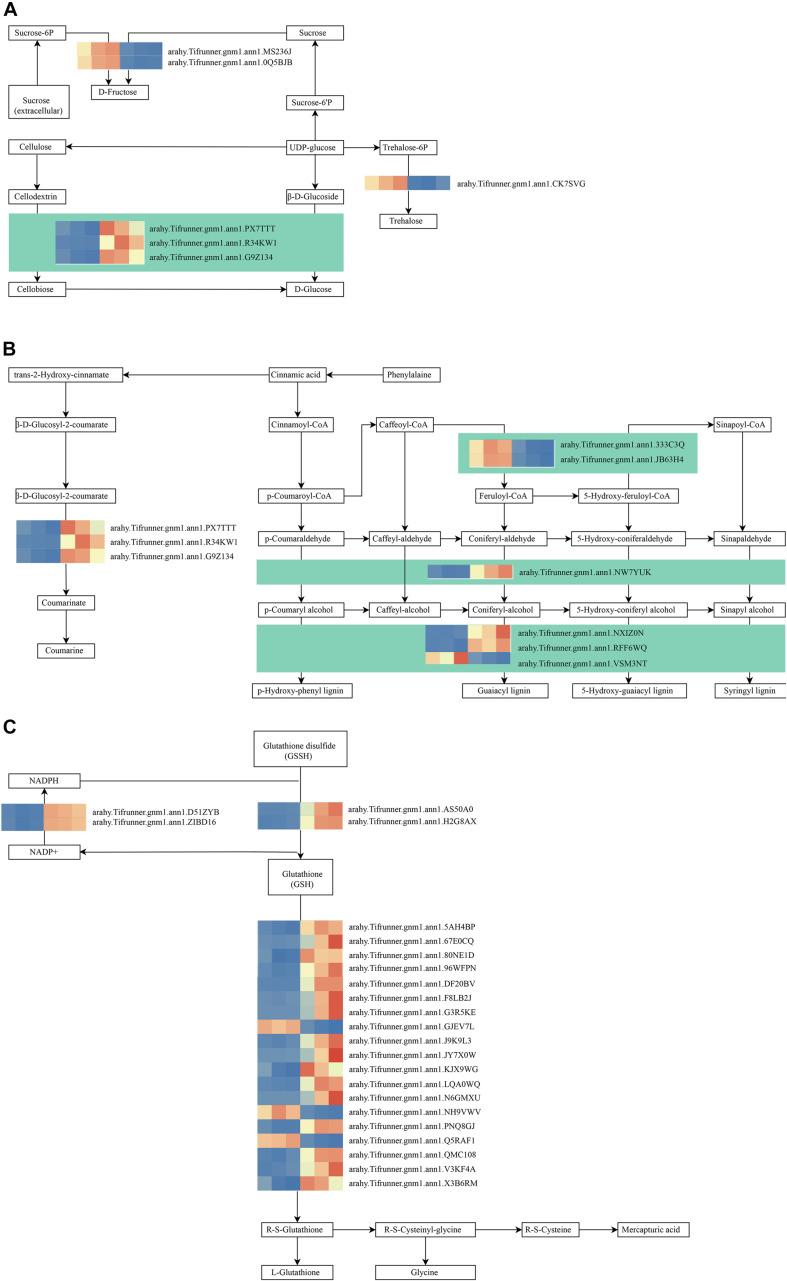
Drought responsive genes in starch and sucrose metabolism **(A)**, flavonoid biosynthesis **(B)**, phenylpropanoid biosynthesis **(B)**, and Glutathione metabolism **(C)** pathways. Relative expression levels are normalized based on the Z-score and shown as a color gradient from low (blue) to high (red). The columns in heat map are 5, 7, and 9 days of well-watered condition, and 5, 7, and 9 days of drought-treated condition under severe drought from left to right, respectively.

**FIGURE 7:**
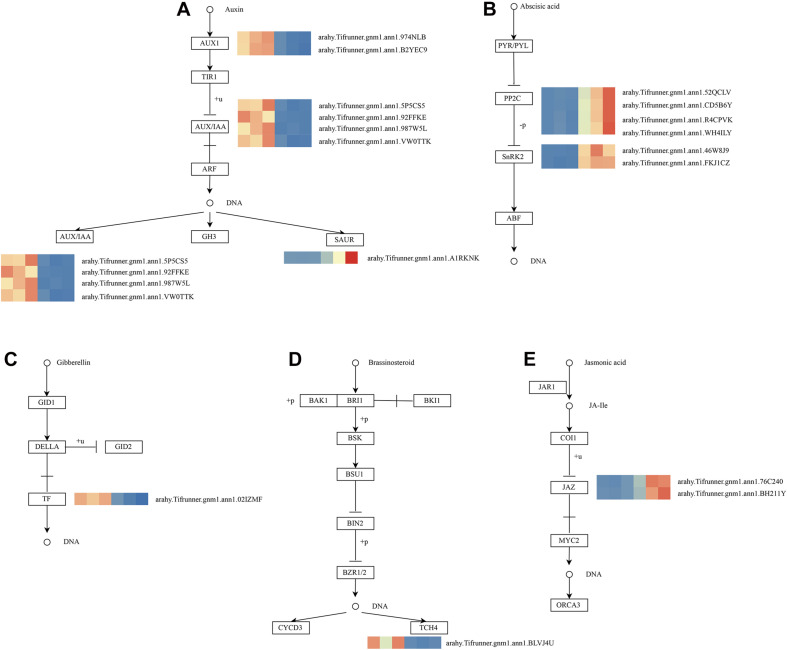
Drought responsive genes in plant hormone signal transduction pathway. **(A)** IAA signal transduction pathway; **(B)** ABA signal transduction pathway; **(C)** GA signal transduction pathway; **(D)** BR signal transduction pathway; **(E)** JA signal transduction pathway. Relative expression levels are normalized based on the Z-score and shown as a color gradient from low (blue) to high (red). The columns in heat map are 5, 7, and 9 days of well-watered condition, and 5, 7 and 9 days of drought-treated condition under severe drought from left to right, respectively.

As shown in Figure 4D, these genes in the MM.darkred module had a similar preponderant expression stage based on the gene expression heatmaps and eigengene histograms. In accordance with the condition of FPKM ≥ 9 for at least one sample, we screened 1,313 common DEGs based on the MM.darkred module and 2,846 common DEGs at three time points under severe drought stress to perform KEGG analysis (Supplementary Figure 2). Multiple crucial pathways involved in plant drought stress were determined, which included “Mitogen activated protein kinase (MAPK) signaling pathway-plant,” “flavonoid biosynthesis,” “starch and sucrose metabolism,” “phenylpropanoid biosynthesis,” “glutathione metabolism,” and “plant hormone signal transduction,” and summarized for analysis (Supplementary Table 3). In MAPK signaling pathway, all seven DEGs were up-regulated throughout the severe drought stress containing two protein kinase superfamily proteins, four protein phosphatase 2C family proteins, and one chitinase family protein (Figure 5). The genes annotated as starch and sucrose metabolism were three down-regulated and three up-regulated (Figure 6A). In total, three down-regulated genes and six up-regulated genes were found in the flavonoid biosynthesis and phenylpropanoid biosynthesis pathways (Figure 6B). The genes annotated as glutathione metabolism exposed to drought stress had three down-regulated genes and 20 up-regulated genes, which were mainly annotated as glutathione S-transferase family proteins (Supplementary Table 3 and Figure 6C). In addition, many genes involved in hormone biosynthesis were detected to be differentially expressed under severe drought stress. A total of 18 DEGs involved in plant hormone signal transduction of auxin (IAA), jasmonic acid (JA), gibberellin (GA), brassinosteroid (BR), and abscisic acid (ABA) metabolism were identified in this study (Figure 7 and Supplementary Table 3). In IAA signal pathway, two AUX1 genes and four AUX/IAA genes were down-regulated under drought at three time points, but only one SAUR gene was up-regulated (Figure 7A). Six up-regulated genes were identified in ABA signal pathway, including four PP2C genes and two SnPK2 genes (Figure 7B). However, only one TF gene and TCH4 gene were down-regulated in the GA and BR signal pathways, respectively (Figure 7C,D). We also found two up-regulated GAZ genes in the SA pathway (Figure 7E). Moreover, the GO terms related to drought response were also identified (Supplementary Figure 2).

### Transcription Factors in Response to Drought Stress

**FIGURE 8:**
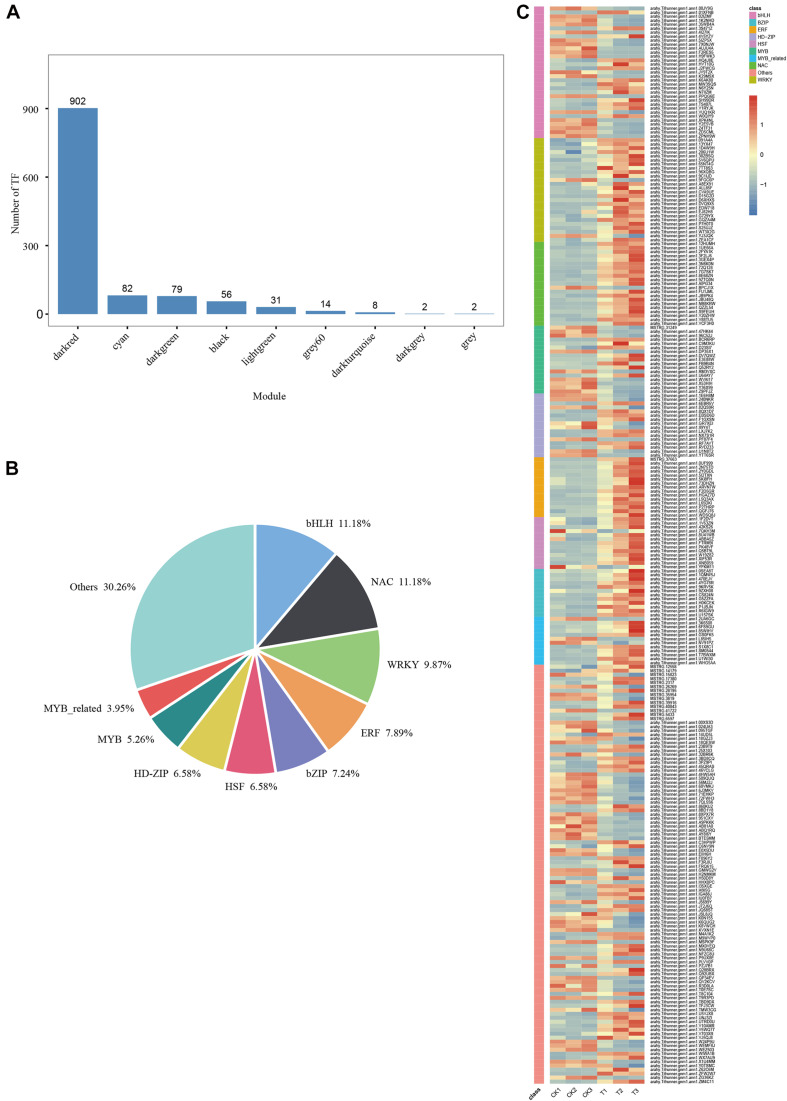
WGCNA of transcription factors analysis in “L422.” **(A)** The number of TFs for each module. **(B)** The proportion of genes in the top 9 abundant TF families in MM.darkred module. **(C)** Heat map of TF gene expression in MM.darkred module. Relative expression levels are normalized based on the Z-score and shown as a color gradient from low (blue) to high (red).

A total of 902 TFs were identified in the MM.darkred module (Figure 8A). One-hundred fifty-two differentially expressed TF genes were obtained according to the condition of FPKM ≥ 9 with at least one sample throughout the severe stress stage (Figure 8C and Supplementary Table 4), of which the TF families of bHLH, NAC, and WRKY were the top three families (Figure 8B). Sixty-one TFs were screened based on the MM.darkred module and 2,846 common DEGs at three time points under severe drought stress (Supplementary Table 4). The key TF genes included *arahy.Tifrunner.gnm1.ann1.PPQG6E* (*BHLH 72*), *arahy.Tifrunner.gnm1.ann1.02IZMF* (*PIL15*), *arahy.Tifr unner.gnm1.ann1.72Q128* (*NAC029*), *arahy.Tifrunner.gnm1. ann1.D15G2D* (*WRKY71*), *arahy.Tifrunner.gnm1.ann1.30ZBSQ* (*WRKY75*), and *arahy.Tifrunner.gnm1.ann1.EDW718* (*WRK Y15*). Overall, the high expression of these TF families in “L422” may play a vital role under severe drought stress.

## Discussion

The cultivated peanut is an allotetraploid (amphidiploid with 2n = 4x = 40) and is relatively drought-tolerant to a certain extent. However, water deficit stress in pod formation stage would seriously affect the yield and productivity of peanuts (Haro et al., 2011; Koolachart et al., 2013). Therefore, improving drought tolerance of peanuts is very important and more research is needed to explore and understand drought stress. Here we performed the physiological and transcriptomic analysis of “L422” at the pod formation stage under drought and well-watered conditions. Then, bioinformatics methods were utilized to analyze differential gene expression in multiple signaling pathways that were potentially associated with drought. The results provided informative clues for the elucidation of drought stress tolerance in peanuts, as well as providing a basis for the identification of drought resistance candidate genes.

### Physiological and Phenotypic Changes of Peanuts in Response to Drought Stress

Plant drought stress response and adaptation are extremely complex, including physiological changes. In this study, we assessed physiological changes of peanuts at the pod formation stage under severe drought conditions. Compared with the control, the phenotypic and physiological changes of “L422” under severe drought stress were obvious (Figure 1). We observed greater leaf rolling with decreasing water content and increasing relative electrical conductivity in drought treated leaves, respectively (Figure, 1B,C). The excessive accumulation of free radicals in cells leads to membrane lipid peroxidation. MDA is the main product of cytoplasmic membrane peroxidation, which is an important index to evaluate plant tolerance to drought stress (Deng et al., 2019). In this study, the MDA content showed that the stressed group was significantly higher than the non-stressed group under severe drought stress. This observation may suggest that the cell membrane of leaves is damaged, which leads to the release of cell membrane lipid and the destruction of membrane structure (Figure 1D). The measurement of RWC, relative electrical conductivity, and MDA confirmed that “L422” suffered physiological damage under severe drought stress. It is well known that drought stress can lead to the accumulation of ROS in plants and its over-accumulation is harmful to plant cells (Blokhina et al., 2003). The scavenging system comprising antioxidants plays important roles in scavenging the ROS. Peroxidase, as an important antioxidant, can minimize cellular damage by scavenging and detoxifying ROS-generated H_2_O_2_ (Sharma et al., 2012). However, we found that POD activity did not change significantly at 9 days of drought stress, which may mean that antioxidant enzymes cannot effectively scavenge ROS under long-term drought conditions (Figure 1G). Various osmoregulatory substances such as soluble sugar and soluble protein can increase the osmotic potential at the cellular level to prevent loss of moisture and enhance plant stress resistance (Shao et al., 2009; Ozturk et al., 2020). In our study, the soluble sugar and soluble protein content increased compared with the control group under severe drought conditions (Figures 1E,F). The result was consistent with former studies (Fu et al., 2011; Ozturk et al., 2020). Also, the soluble protein content showed a greater increase at 7 days of drought stress, but the subsequent changes were not significant. The greater increase may be due to the expression of new stress proteins, and then the increase was not significant because of the serious decline in photosynthesis. These physiological and phenotypic changes suggest that severe drought stress has a serious effect on the pod formation stage of peanuts.

### Analysis of Starch and Sucrose Metabolism in Response to Drought Stress

Based on KEGG pathway enrichment analysis, starch and sucrose metabolism pathway was identified in the MM.darkred module. The starch and sucrose metabolic process is widely identified in many plants under drought stress (Min et al., 2016; Ma et al., 2017; Khan et al., 2019; Hong et al., 2020). Trehalose, glucose, and sucrose are important soluble sugars to maintain cell osmotic potential (Konigshofer and Loppert, 2015; Han et al., 2016; Cui et al., 2019). Based on the analysis of soluble sugar content in the present study (Figure 1E), we hypothesized that these DEGs were involved in the biosynthesis of trehalose, glucose, and sucrose to maintain cell osmotic potential under severe drought stress. Several studies have highlighted the role of related genes in drought stress. For instance, overexpression of *OsTPS1* (trehalose-6-phosphate synthase) increased the amount of trehalose and proline, and enhanced abiotic stress tolerance in plants (Li et al., 2011). Simultaneously, Invertases (INVs) plays an important role in primary metabolism and plant development, which can hydrolyze sucrose into glucose and fructose (Ruan et al., 2010; Min et al., 2016) and contribute to osmotic adjustment under water deficit conditions (Konigshofer and Loppert, 2015). A similar result has shown that starch and sucrose metabolism was significantly affected by drought stress in peanut (Gundaraniya et al., 2020). These results showed genes involved in the regulation of starch and sucrose metabolism may play an important role in drought stress.

### Analysis of Secondary Metabolites Biosynthesis and Glutathione Metabolism in Response to Drought Stress

Biosynthesis of secondary metabolites such as phenylpropanoids and flavonoids is essential for a plant’s response to stresses (Hernandez et al., 2009; Nakabayashi et al., 2014; Deng and Lu, 2017; Sharma et al., 2019). In our study, two caffeoyl-CoA 3-O-methyltransferase (CCoAOMT) related genes were down-regulated. *CCoAOMT1* and *COMT1* have a vital role in the biosynthesis of lignin, flavonoids, and sinapoyl malate in *Arabidopsis* (Do et al., 2007). Moreover, POD-encoding genes were also induced under severe drought stress. We speculated that these genes may participate in the regulation of peanut response to drought by combining the result of POD activity. Glutathione metabolism plays a key role in cellular defense (Noctor et al., 2002; Ball et al., 2004). In glutathione metabolism, glutathione can be oxidized to glutathione disulfide, and glutathione disulfide is again reduced to glutathione by glutathione reductase (Gong et al., 2018; Borgohain et al., 2019). And, dehydroascorbate (DHA) is reduced to ascorbic acid (Asc) by dehydroascorbate reductase (DHAR) in the presence of reduced glutathione, which in turn is regenerated by glutathione reductase (Chang et al., 2017). In our study, several key enzymes involved in glutathione metabolism were identified, which were also reported to be involved in drought tolerance regulation in *Oudneya* (Talbi et al., 2015). Consistently, Overexpression of *JcDHAR* can effectively enhance the tolerance to oxidative stress in plants (Chang et al., 2017). Therefore, the regulation of glutathione metabolism might contribute to drought tolerance in peanut under severe drought stress.

### Analysis of Plant Hormone Signal Transduction and Protein Kinases in Response to Drought Stress

Multiple hormone-related pathways have been reported to be involved in the drought tolerance of plants. In this study, seven genes encoding protein involved in IAA signaling pathway were differentially expressed, including AUX1s, AUX/IAAs, and SAUR, under severe drought stress. A previous study has also demonstrated that overexpression of *OsIAA6* increased in transgenic rice drought tolerance (Jung et al., 2015). In another case, *TaSAUR75* transgenic Arabidopsis showed higher root length and survival rate under salt and drought stress (Guo et al., 2018). Typically, environmental stress is known to trigger changes in ABA levels and ABA regulates plant defense to drought stress (Verma et al., 2016). The central signaling complex PYR/PYL (Pyracbactin Resistance/Pyracbactin Resistance-like)-PP2Cs (Protein Phosphatase 2C)-SnRK2s (SNF1-Related Protein Kinases type 2) of ABA signaling pathway was activated in “L422.” Among them, the genes encoding protein PP2Cs and SnRK2s were up-regulated but did not affect PYR/PYL. JA signaling pathway is associated with the alleviation of drought stress (Ali and Baek et al., 2020; Wang et al., 2020). Here, we found that two JAZ genes associated with JA signal transduction were predominantly expressed in “L422.” A study found that OsJAZ1 could act as a transcriptional regulator of the OsbHLH148-related JA signaling pathway, leading to drought tolerance (Seo et al., 2011). Additionally, we found down-regulated *XTH23* and *PIL* genes in GA and BR signaling pathways, respectively. Interestingly, a previous study has reported that *XTH23* was induced from seed priming with BR on peanut under drought condition (Huang et al., 2020). Based on the analysis results, these DEGs may play a vital role via hormonal crosstalk in response to drought. Moreover, signaling pathways are induced under environmental stresses, in which one of the major pathways is MAPK cascade in plant. MAPK cascade can convert environmental signals into molecular and cellular responses (Kaur and Gupta, 2005; Cheong and Kim, 2010; Sinha et al., 2011; Kumar et al., 2020). Previous findings clearly demonstrated that MAPK cascades were implicated in ABA and ethylene (ET) signaling (Zwerger and Hirt, 2001; Kar, 2011; de Zelicourt et al., 2016; Jagodzik et al., 2018). Interestingly, our study found that seven DEGs were detected in the ET and ABA signaling pathways. These genes will provide important implications for further research on the drought tolerance of peanuts.

### Major TFs Involved in the Drought Response of Peanuts

Transcription factors as key regulators of transcription are important in plant responses to drought stress. In the present study, the bHLH family contained the most members, followed by NAC and WRKY families, indicating that they played an important role in coping with drought stress. Many transcription factors have been demonstrated to play an important role under drought stress in many crops. For example, a previous study reported that MdbHLH130 acts as a positive regulator of drought stress responses through modulating stomatal closure and ROS-scavenging in tobacco (Zhao Q. et al., 2020). Further, a recent study has shown that some *NAC* genes were induced under salt and drought stresses via RNA-seq and RT-qPCR analysis in peanut (Yuan et al., 2020). Interestingly, *AhNAC 65*, *AhNAC* 87, and *AhNAC 102* were induced in both drought and salt stresses, which were up-regulated in our result. We speculated that the three genes may play an important role in stress resistance. Although *NAC* 18 was only induced in salt stress, it was up-regulated in our result. Therefore, further studies of key NACs in our result will help to reveal the role of NACs in drought resistance in peanut. In soybean plant, GmWRKY54 conferred drought tolerance in transgenic soybean enhancing ABA/Ca^2+^ signaling pathways for stomatal closure and activating the expression of large numbers of stress-related TFs (Wei et al., 2019). Here, we found that 15 differentially expressed WRKYs were all up-regulated in the present study. Additionally, three (arahy.Tifrunner.gnm1.ann1.D15G2D, arahy. Tifrunner.gnm1.ann1.30ZBSQ, and arahy.Tifrunner.gnm1.ann1. P7H0T0) of 15 WRKYs were also induced and the rest of the WRKYs did not change in our previous study (Supplementary Table 4) (Zhao Q. et al., 2020). The roles of these WRKYs need to be elucidated in further investigation. Taken together, these differentially expressed TFs might be involved in response to drought stress, and they would provide important information for the study of drought tolerance in peanut.

## Conclusion

In this study, we performed the physiological and transcriptomic analysis of “L422” at the pod formation stage under drought and well-watered conditions. Many DEGs were identified between well-watered and drought conditions by using RNA-Seq and WGCNA. The DEGs related to “MAPK signaling pathway-plant,” “flavonoid biosynthesis,” “starch and sucrose metabolism,” “phenylpropanoid biosynthesis,” “Glutathione metabolism,” and “plant hormone signal transduction” were enriched in drought-tolerant cultivar. And numerous TF genes participated in the regulation networks under drought stress. The results provided a basis for further research on drought resistance genes in peanut.

## Data Availability Statement

The datasets presented in this study can be found in online repositories (BioProject ID: PRJNA706902).

## Author Contributions

LL and NZ designed the study and wrote the manuscript. NZ carried out the experiments and analyzed data. LL revised the manuscript. SC, BL, and HD carried out the field experiments for screening material. XL, YL, MH, XY, and GM assisted in writing the manuscript. All authors contributed to the manuscript and approved the final manuscript to publish.

## Conflict of Interest

The authors declare that the research was conducted in the absence of any commercial or financial relationships that could be construed as a potential conflict of interest.

## Supplementary Material

Supplementary Figure 1 Phenotypic and physiological changes of four cultivars with different drought tolerance under drought stress. **(A)** Phenotypic responses of four cultivars to drought stress. The changes of the relative water content **(B)** and relative electric conductivity **(C)** in leaves of four cultivars under well-watered and drought conditions. Values are the mean ± standard deviation of three biological replicates.

Supplementary Figure 2 *Q*-value heatmap of the GO significant pathway enrichment of the three main ontology for the 1,313 common DEGs in the MM.darkred module. The color scale indicates the Q-value.

Supplementary Table 1 Sequences of the primers used in this study.

Supplementary Table 2 Detailed information on the obtained reads *via* RNA-Seq.

Supplementary Table 3 DEGs of six vital metabolic pathways in the MM.darkred module.

Supplementary Table 4 Differentially expressed TFs in the MM.darkred module and differentially expressed WRKY TFs in previous study.
