# Supplementary figures and images for: Transcriptome and Co-expression Network Analyses Reveal Differential Gene Expression and Pathways in Response to Severe Drought Stress in Peanut (Arachis hypogaea L.)
Source: Front Genet. 2021 Apr 30;12:672884. doi: 10.3389/fgene.2021.672884 (PMC8120245; doi:10.3389/fgene.2021.672884)

A

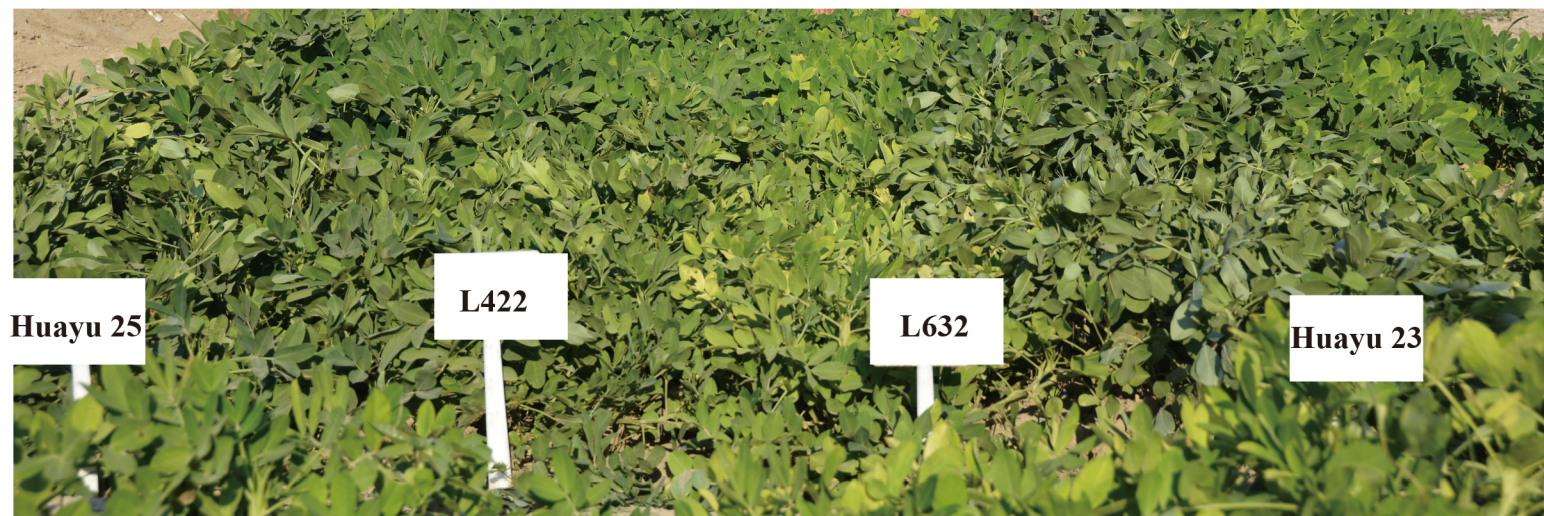

B

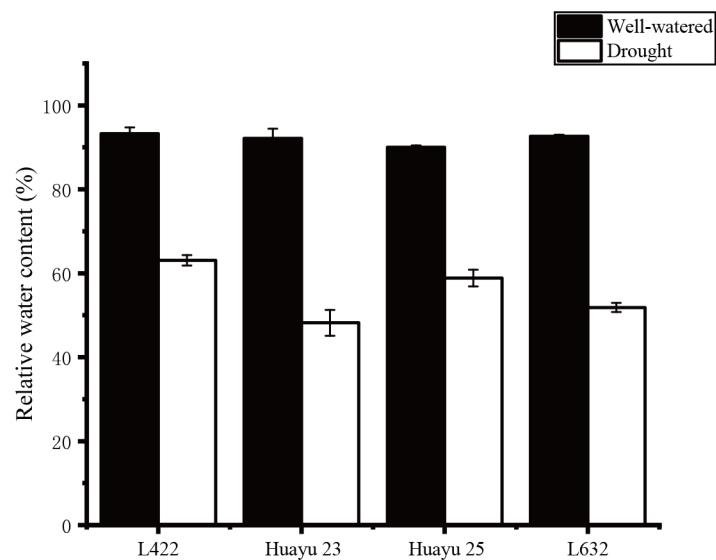

C

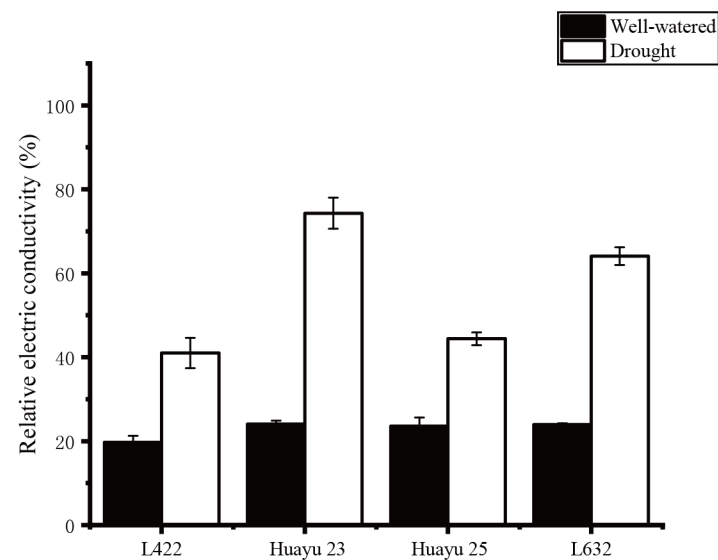

Supplement: Supplementary Figure 1 — Phenotypic and physiological changes of four cultivars with different drought tolerance under drought stress. (A) Phenotypic responses of four cultivars to drought stress. The changes of the relative water content (B) and relative electric conductivity (C) in leaves of four cultivars under well-watered and drought conditions. Values are the mean ± standard deviation of three biological replicates. [file Data_Sheet_1.PDF]

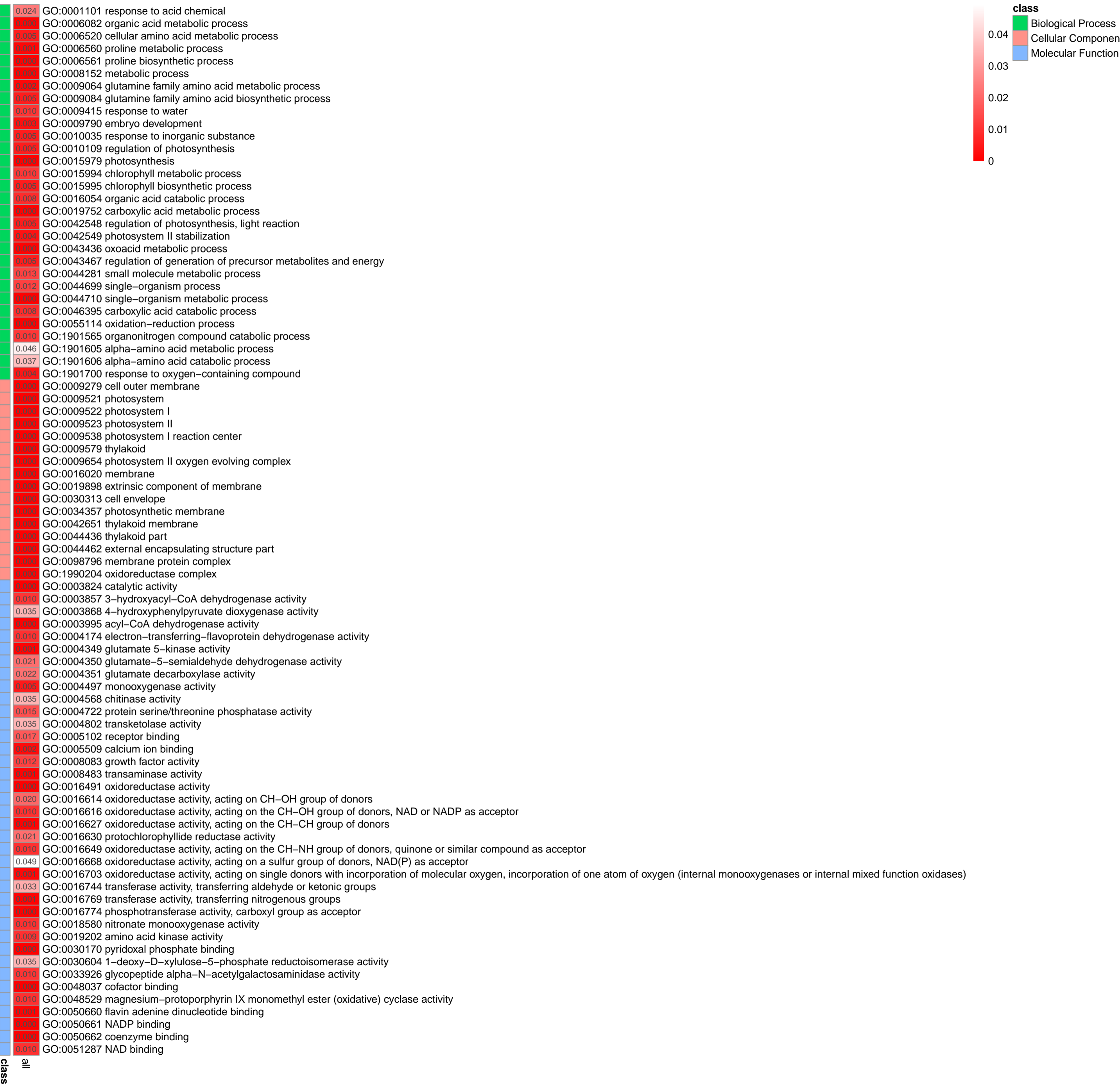

Supplement: Supplementary Figure 2 — Q-value heatmap of the GO significant pathway enrichment of the three main ontology for the 1,313 common DEGs in the MM.darkred module. The color scale indicates the Q-value. [file Data_Sheet_2.PDF]
